# The clinical relevance of sleep disturbance (“insomnia”) in patients with advanced cancer receiving palliative care: a scoping review

**DOI:** 10.1007/s00520-026-10738-3

**Published:** 2026-05-07

**Authors:** Shauna Munir, Eva Jones, Faith Precious Omeokwe, Andrew Neil Davies

**Affiliations:** 1 Our Lady’s Hospice & Care Services (Community Palliative Care), Dublin, Ireland; 2 School of Nursing, University College Dublin, Midwifery & Health Systems, Dublin, Ireland; 3 St. James’ Hospital, Dublin, Ireland; 4 School of Medicine, Trinity College Dublin, Dublin, Ireland; 5 School of Nursing, George Washington University, Ashburn, VA USA; 6 School of Medicine, University College Dublin, Dublin, Ireland

**Keywords:** Sleep disturbance, Sleep initiation and maintenance disorders, Palliative care, Hospice care, Clinical features

## Abstract

**Purpose:**

Sleep disturbance (“insomnia”) is common in patients with advanced cancer receiving specialist palliative care. The aim of this scoping review was to determine the clinical relevance of sleep disturbance in this cohort of patients.

**Methods:**

Standard methodology was employed, and four databases were searched from inception (Medline, CINAHL, Embase, and APA PsycInfo). Hand searching of relevant sources was also undertaken. Included studies needed to have a sleep focus, and to utilise a validated sleep assessment tool (and/or objective measure of sleep quality).

**Results:**

Sixteen studies met the criteria for inclusion. The studies highlight that sleep disturbance is associated with a range of physical symptoms (e.g. fatigue, drowsiness), a variety of psychological problems (e.g. anxiety, depression), impaired quality of life, and reduced overall survival.

**Conclusion:**

Sleep disturbance is an “orphan” symptom, and the results of this scoping review suggest that it deserves much greater attention. Indeed, healthcare professionals should screen all palliative care patients for the problem and, when identified, perform a thorough assessment and initiate an appropriate treatment.

## Introduction

Sleep disorders have been divided into six major groups by the American Academy of Sleep Medicine (AASM): (1) insomnia disorders; (2) sleep-related breathing disorders; (3) central disorders of hypersomnolence; (4) circadian rhythm sleep–wake disorders: (5) parasomnias; and (6): sleep-related movement disorders [1]. An insomnia disorder is defined as “a complaint of trouble initiating or maintaining sleep which is associated with day-time consequences and is not attributable to environmental circumstances or inadequate opportunity to sleep” [2]. The AASM specifies three subtypes of insomnia disorder based upon the presence/duration of relevant diagnostic criteria: (a) “short-term” insomnia disorder (< 3 months); (b) “chronic” insomnia disorder (≥ 3 months; ≥ 3 times a week); and “other” insomnia disorder, i.e. short term/chronic insomnia disorder diagnostic criteria unfulfilled. [1]. Importantly, non-sleep medicine research studies seldom use this classification/these diagnostic criteria, and palliative care research studies generally use the term “sleep disturbance” to describe analogous problems [3].

Sleep disturbance is common in all groups of patients with cancer but appears to be particularly common in patients with advanced cancer. Thus, the reported combined prevalence of “sleep disturbance” is 60.7% (95% confidence interval: 58.1–63.3%) overall, and 70.8% (95% confidence interval: 61.7–78.5%) in patients with advanced cancer [3]. Numerous factors have been linked to the development/persistence of sleep disturbance in cancer patients, including the underlying cancer [4], anticancer treatments [5], supportive care treatments (e.g. corticosteroids, opioids) [6], and uncontrolled symptoms (both physical and psychological) [7]. Importantly, sleep disturbance is also common amongst the family carers of patients with advanced cancer [8], and it is highly likely that the family carers with sleep disturbance are negatively impacting the sleep of the patient (and vice versa).

Despite its significant prevalence, sleep disturbance has been identified as an “orphan” symptom [9], i.e. one of the “symptoms not regularly assessed in clinical practice, and consequently little studied and not properly treated” [10]. The aim of this scoping review is to determine the clinical relevance of sleep disturbance (“insomnia”) in patients with advanced cancer receiving palliative care. The review will not address sleep disturbance in patients with advanced non-malignant disease, although this appears to be a common problem in this cohort [11]. Similarly, the review will not address other sleep disorders in patients with advanced cancer, although some appear to be common in this cohort (e.g. sleep-related breathing disorders, circadian rhythm sleep–wake disorders) [12, 13].

## Methods

Standard methodology was used to perform the scoping review [14, 15], and to report the results of the scoping review (i.e. PRISMA Extension for Scoping Reviews) [16].

### Search strategy

The final search was performed in October 2025. A detailed search strategy was developed for Medline (Appendix 1), and adapted as needed for CINAHL, Embase, and APA PsycInfo. All databases were searched from inception. Non-English studies were excluded from the review. Conference abstracts were also excluded from the review.

### Study eligibility criteria

Studies needed to involve adult (> 18 yr) patients with “advanced cancer” [17] that were receiving “specialist palliative care” (as either an outpatient or an inpatient) [18]. Additionally, studies needed to have a sleep focus, and utilise a validated sleep assessment tool (rather than a generic symptom assessment tool, or quality-of-life assessment tool). Studies involving mixed populations could be included as long as there was separate reporting of results for patients with advanced cancer. Case reports, review articles, and other records without original information were excluded.

### Data management and synthesis

EndNote 20™ software (Clarivate Analytics LLP, USA) was used to store the retrieved articles, and Covidence software (Veritas Health Innovation, Australia) was used to screen these retrieved articles.

All the reviewers separately screened the titles and abstracts for full-text articles to retrieve/appraise. Conflicts were resolved by consensus. Two reviewers (SM, EJ) separately appraised the full-text articles and extracted the relevant information using a review-specific template (i.e. study ID, study population, study methodology, study results). Another reviewer (AD) was available to resolve conflicts.

The reference lists of all retrieved full-text articles, relevant chapters in major palliative care textbooks, and relevant sections of major palliative care guidelines were hand searched (SM, EJ) for other potential studies.

## Results

**Fig. 1:**
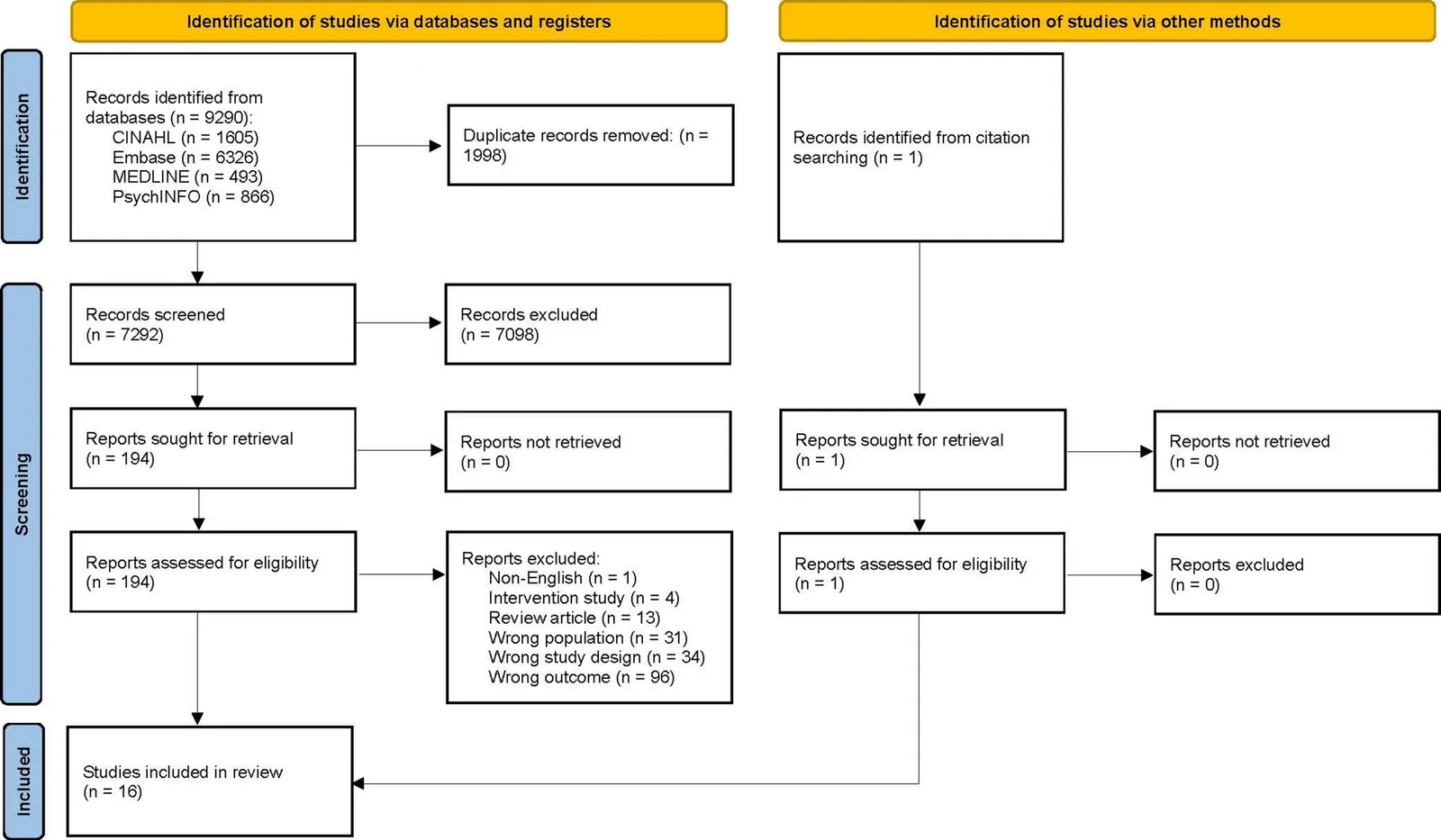
PRISMA flow diagram

The literature review identified a number of studies relating to sleep disturbance in patients with advanced cancer and/or receiving specialist palliative care (see Fig. 1), but only 16 studies met the inclusion criteria for the scoping review (see Table 1) [6, 19–33]. All studies were observational, and the focus was on clinical features (rather than treatment).

**Table 1:** Included studies

Study	Study population	Methodology	Study results
Bernatchez et al., 2020 [20] Canada	*n* = 57 *“community-dwelling patients with cancer receiving palliative care”* Cancer diagnoses (commonest): lung (17.5%), urinary & gastrointestinal (17.5%), gynaecological (10.5%), haematological (10.5%) Mean age = 65.8 yr ± 10.8 Female = 47.4%	Insomnia Severity Index (ISI) Epworth Sleepiness Scale (ESS) Sleep diary Physical Symptoms Questionnaire (PSQ) Functional Assessment of Chronic Illness Therapy-Fatigue Scale (FACIT-FS) Hospital Anxiety and Depression Scale (HADS) Missoula-VITAS Quality of Life Index (MVQOLI) Actigraphy	ISI score correlated with ESS score, pain, PSQ composite score, fatigue, MVQOLI global score, anxiety, and depression Actigraphy: Lower sleep efficiency correlated with pain, and depression Sleep onset latency correlated with gastrointestinal symptoms Awakenings correlated with gastrointestinal symptoms Time in bed correlated with PSQ composite score, and gastrointestinal symptoms
Jakobsen et al., 2020 [21] Norway	*n* = 41 (analysed = 40) *“patients hospitalized for symptom control at a specialized inpatient unit”* Cancer diagnoses: gastrointestinal (55%), prostate (20%), lung (5%), skin (5%), urological (5%) Median age = 70 yr (range 46–91 yr) Female = 40%	Pittsburgh Sleep Quality Index (PSQI)—total score > 5 = “poor sleep” Karolinska Sleepiness Scale Sleep diary Actigraphy (Polysomnography)	46%—“poor sleep” Actigraphy: Moderate correlation between sleep diary and actigraphy for total sleep time, and sleep efficiency (but not other parameters)
Saricam et al., 2019 [22] Turkey	*n* = 192 Cancer diagnoses: gastrointestinal (39.1%), lung (24%), head & neck (14.1%) Mean age = 68.4 yr ± 14.1 Female = 43.8%	Athens Insomnia Scale (AIS)—total score > 6 = sleep disorder	27.1%—sleep disorder
Bernatchez et al., 2018 [23] Canada	*n* = 51 *“community dwelling cancer patients receiving palliative care”* Cancer diagnoses: urinary & gastrointestinal (19.6%), lung (15.7%), breast (9.8%) Mean age = 66.4 yr ± 10.5 Female = 49%	“Relevant sections” of Duke Structured Interview for Sleep Disorders—“insomnia disorder” diagnosed if meeting DSM-5 criteria; “insomnia symptoms” if not meeting DSM-5 criteria Sleep Diary Actigraphy	21.6%—insomnia disorder 9.8%—insomnia symptoms 21.5%—hypersomnolence disorder 3.9%—sleep apnoea syndrome/symptoms 2%—restless legs syndrome
Davies et al., 2017 [24] United Kingdom	*n* = 174 *“inpatients and outpatients receiving specialist palliative care”* Cancer diagnoses: gastrointestinal (35.5%), breast (16%), urological (14%) Median age = 66 yr (range 35–90 yr) Female = 57.5%	PSQI—total score > 5 = “poor sleep quality”	70.5%—poor sleep quality Poor sleep quality correlated with younger age, inpatient setting, vivid dreams, and nightmares
Mercadante et al., 2017 [25] Italy	*n* = 219 *“patients with advanced cancer admitted to an acute palliative-supportive care unit”* Cancer diagnoses: gastrointestinal (26%), lung (22.8%), breast (16.5%) Mean age = 65.4 yr ± 12.4 Female = 49.3%	AIS—total score > 6 = sleep disturbance [“Mild”—AIS 6–8; “moderate”—AIS 9–12; “intense”—AIS 13–16; “maximum”—AIS 17–24] Edmonton Symptom Assessment System (ESAS) HADS	100%—sleep disturbance [Mild—0.5%; moderate—15.5%; intense—26.5%; maximum—57.5%] AIS scores were correlated with poor performance status, pain, tiredness, drowsiness, anorexia, feeling of wellbeing, anxiety, depression, and use of drugs (i.e. corticosteroids, benzodiazepines) [Only anxiety, and depression were correlated in multivariate analysis]
Yennurajalingam et al., 2017 [26] USA	*n* = 180 *“advanced cancer patients” with “sleep disturbance of* ≥ *1/10 on a 0–10 scale”* Cancer diagnoses: gastrointestinal (19%), lung (18%), genitourinary (17%) Median age = 57 yr Female = 51%	PSQI—total score ≥ 5 = “sleep disturbance” Insomnia Severity Index (ISI) ESS STOP-Bang Scoring Model—obstructive sleep apnoea tool Screening for restless leg syndrome ESAS HADS	62%—sleep disturbance 61%—obstructive sleep apnoea 38%—restless legs syndrome Sleep disturbance correlated with pain, feeling of well-being, shortness of breath, and obstructive sleep apnoea
Mercadante et al., 2015 [6] Italy	*n* = 820 *“patients with advanced cancer* *admitted to different palliative care settings (oncology, home care, palliative care unit, or hospice)”* Cancer diagnoses: lung (22.4%), gastrointestinal (31.6%), breast (10.7%) Mean age = 69.7 yr ± 12.7 Female = 47.7%	AIS—total score > 6 = sleep disturbance ESAS HADS	78%—sleep disturbance AIS scores were correlated with younger age, cancer diagnosis (i.e. lung, gastrointestinal, breast, head & cancer), poor performance status, anxiety, depression, and use of drugs (i.e. hormone therapy, corticosteroids, opioid analgesics)
Mercadante et al., 2021 [19] Italy (Secondary/subgroup analysis of Mercadante et al., 2015)	*n* = 182 *“patients with advanced cancer in different palliative care settings”* Cancer diagnoses: lung cancer (100%) Mean age = 69.9 yr ± 10.8 Female = 33.5%	AIS—total score > 6 = sleep disturbance ESAS HADS	83.2%—sleep disturbance Sleep disturbance correlated with poor performance status, pain, nausea, drowsiness, anxiety and depression
Davis et al., 2014 [27] USA	*n* = 715 *“patients with cancer referred to the palliative medicine program inpatient, outpatient, and consultation service”* Cancer diagnoses: % not stated Median age = 64 yr (range 29—91 yr) Female = % not stated	Screening question: Do you have problems getting to sleep, staying asleep, or waking up early? ISI—score 15—28 = clinical insomnia	14%—positive response screening question had sleep problems 9%—clinical insomnia Clinical insomnia correlated with pain, tiredness, and depression ISI score correlated poorly with amount of sleep
Delgado-Guay et al., 2011 [28] USA	*n* = 101 *“had a diagnosis of advanced cancer and had been referred to the…palliative care clinic”* Cancer diagnoses: lung (21%), breast (20%), gastrointestinal (15%) Median age = 60 yr (range 25–84 yr) Female = 52%	PSQI—total score ≥ 5 = “poor sleeper” ESAS	85%—“poor sleeper” Poor sleep correlated with pain, feeling of wellbeing, anxiety, and depression
Mystakidou et al., 2007a [31] Greece	*n* = 102 *“terminally ill cancer patients who were referred to the Pain Relief and Palliative Care Unit”* Cancer diagnoses: urogenital (23.5%), lung (22.5%), breast (21.6%), gastrointestinal (21.6%) Mean age = 62.8 yr Female = 54.9%	PSQI (Greek version)—total score > 8 = “poor sleeper” Greek Brief Pain Inventory Beck Depression Inventory (Greek version) Beck Hopelessness Scale Greek Schedule of Attitudes toward Hastened Death Short Form 12 Health Survey (SF-12)—health related quality of life tool that gives a mental component summary score (MCS), and physical component summary score (PCS)	73.5%—“poor sleeper” Poor sleep correlated with “interference of pain with mood", hopelessness, and use of opioid analgesics
Mystakidou et al., 2007b [32] Greece (secondary analysis of Mystakidou et al., 2007)	See above	See above PSQI (Greek version)—total score ≥ 5 = “poor sleeper”	Poor sleep correlated with pain scores, MCS/PCS scores (i.e. worse quality of life), and use of opioid analgesics
Mystakidou et al., 2007c [33] Greece (secondary analysis of Mystakidou et al., 2007)	See above	See above	Poor sleep correlated with desire for hastened death (as was use of sleeping medication)
Mystakidou et al., 2009a [29] Greece (secondary analysis of Mystakidou et al., 2007a)	See above	See above	Poor sleep correlated with depression, and hopelessness
Mystakidou et al., 2009b [30] Greece (appears to be subgroup analysis of Mystakidou et al., 2007a)	*n* = 82 *“patients referred to the (palliative care) unit”* Cancer diagnoses: urogenital (26.8%), gastrointestinal (22.0%), breast (20.7%), lung (20.7%) Mean age = 62.6 yr ± 13.7 Female = 56.1%	PSQI (Greek version)—total score ≥ 5 = “poor sleeper” Greek Brief Pain Inventory Beck Depression Inventory (Greek version) Beck Hopelessness Scale SF-12 Impact of Events Scale-Revied – PTSD assessment tool	96.3%—“poor sleeper” Poor sleep correlated with pain scores, MCS/PCS scores, depression, hopelessness, and PTSD,

### Epidemiology

Sleep disturbance appears to be a generic problem in patients with cancer, including those with sarcomas [34], and those with haematological malignancies [35]. The median prevalence in the included studies was 72% (range 9 to 100). Sleep disturbance is more common in patients with a poor performance status [6, 25, 32]. Several studies reported a higher prevalence in younger patients [6, 24, 36, 37], although a previous review suggested the opposite [7].

### Clinical features—physical problems

Studies report a range of clinical features, including difficulty initiating sleep, and difficulty maintaining sleep (waking during night, waking too early) [38, 39]. Additionally, there is an association with daytime sleepiness [20, 40], vivid dreams [24], and nightmares [24]. They also report varying associations between physical problems and sleep disturbance, although the nature of the relationships is impossible to determine: the problems include fatigue [20, 41], “tiredness” [25, 27], pain [19, 20, 25–28, 32], (absence of) feeling of wellbeing [25, 26, 28, 42], drowsiness [19, 25], and delirium [43, 44].

### Clinical features—psychological problems

Similarly, studies report varying associations between psychological problems and sleep disturbance: the problems include “psychosocial distress” [36, 45], anxiety [6, 20, 25, 28, 46], depression [6, 20, 25, 27–29], hopelessness [29, 31], desire for hastened death [33, 47], and even suicidal ideation [48].

### Clinical features—other issues

Additionally, studies involving palliative care populations have reported an association with impaired spiritual wellbeing (i.e. “meaning”) [49]. Unsurprisingly, given the above, sleep disturbance is associated with impaired quality-of-life [20, 30, 32]. Furthermore, the importance of sleep disturbance to quality-of-life appears to increase closer to death [50, 51]. Indeed, sleep disturbance is associated with a worse prognosis [52, 53].

Some studies have reported “symptom clusters” including sleep disturbance with pain and depression [54], and sleep disturbance with anxiety and depression [55]. However, other studies have not confirmed these symptom clusters [56], and their existence as a discrete entity has been recently questioned [54].

### Objective measures

Only three studies involved objective assessments of sleep [20, 21, 23], with all three using actigraphy and one attempted utilising polysomnography (with very limited success) [21]. It appears that there is often a mismatch between subjective opinions and objective measures of sleep disturbance [21, 57].

## Discussion

This scoping review highlights that sleep disturbance is common in patients with advanced cancer receiving palliative care, and that sleep disturbance is a major cause of morbidity in this cohort of patients. Sleep disturbance is associated with a number of physical symptoms, and these may either precipitate and/or perpetuate the problem. In either scenario, the sleep disturbance may go on to exacerbate the physical problem (and the patient’s ability to cope with the physical problem). The same is true for psychological problems. Importantly, this scoping review highlights that many patients have concomitant physical and psychological problems.

The scoping review identified 16 studies that met the criteria for inclusion. However, the literature contains many more studies of sleep disturbance in this cohort of patients. Indeed, further observational studies of this clinical problem appear unwarranted, unless the focus of the study is to answer one of the outstanding clinical issues (see below), or to investigate pharmacological, and especially non-pharmacological, interventions for sleep disturbance in this cohort of patients (since there is currently a paucity of evidence to guide clinical practice) [58].

The outstanding clinical issues are (a) understanding/overcoming the “orphan” symptom status of sleep disturbance; (b) determining the relationship between sleep disturbance and physical/psychological problems (and how to best manage concomitant problems); (c) determining the relationship between objective and subjective measures; and d) determining the relevance of objective measures (versus subjective measures). Thus, further research is needed to confirm/refute reported disparities between objective and subjective measures, and (if so) whether or not the identified disparities are of any clinical relevance in this cohort of patients.

Improving subjective sleep disturbance is clearly essential, especially within the palliative care population. However, improving objective sleep disturbance may be relevant for specific clinical outcomes (e.g. quality-of-life, survival) [13]. Importantly, the relevant evidence base for pharmacological (e.g. benzodiazepines, melatonin) and non-pharmacological interventions (e.g. cognitive behavioural therapy for insomnia/CBT-I, exercise) is extremely limited [58, 59], and so further research is also required to determine the optimal treatment(s) in this cohort of patients.

The strengths of this scoping review are the methods employed, including the broad search of the literature (without time limits). A limitation is the exclusion of non-English studies and the restriction to patients with advanced cancer receiving specialist palliative care. A further limitation is the paucity of studies reporting objective measures of sleep disturbance in this cohort of patients.

In conclusion, sleep disturbance is common in patients with advanced cancer receiving palliative care and is a major cause of morbidity and impaired quality of life in this cohort of patients. Healthcare professionals need to screen all patients for the problem and, when identified, perform a thorough assessment and initiate appropriate (individualised) treatment.

## Data Availability

No datasets were generated or analysed during the current study.

## Appendix 1

Medline search strategy

1. SLEEP – MeSH or 2–13
2. SLEEP DEPRIVATION – MeSH
3. SLEEP DISORDERS, CIRCADIAN RHYTHM – MeSH
4. SLEEP DURATION – MeSH
5. SLEEP INITIATION AND MAINTENANCE DISORDERS – MeSH
6. SLEEP LATENCY – MeSH
7. SLEEP QUALITY – MeSH
8. SLEEP WAKE DISORDERS – MeSH
9. SLEEP AROUSAL DISORDERS – MeSH
10. SLEEP DISORDERS, INTRNSIC – MeSH
11. INSOMNIA – Keyword
12. SLEEP DISTRUBANCE – Keyword
13. SLEEP PROBLEM – Keyword
14. and PALLIATIVE CARE – MeSH or
15. HOSPICE CARE – MeSH
16. TERMINAL CARE – MeSH
17. END OF LIFE CARE – Keyword
